# Thickness‐Dependent Creep in Lithium Layers of All‐Solid‐State Batteries under Stack Pressures

**DOI:** 10.1002/advs.202517361

**Published:** 2025-11-16

**Authors:** Chuangchuang Duan, Yiming Feng, Tianliang Lin, Ruifang Ye, Mingqiang Li, Jici Wen, Chunguang Chen, Yujie Wei

**Affiliations:** ^1^ College of Mechanical Engineering and Automation Huaqiao University Xiamen 361021 China; ^2^ Fujian Key Laboratory of Green Intelligent Drive and Transmission for Mobile Machinery Huaqiao University Xiamen 361021 China; ^3^ LNM Institute of Mechanics Chinese Academy of Sciences Beijing 100190 China; ^4^ School of Engineering Sciences University of Chinese Academy of Sciences Beijing 100049 China

**Keywords:** all‐solid‐state batteries, diffusion, lithium metal, power‐law creep, stack pressure

## Abstract

Stack pressure is broadly explored in improving contact at the lithium metal–solid‐state electrolyte interface of all‐solid‐state lithium‐metal batteries (ASSLBs). The effectiveness of this procedure relies heavily on the time‐dependent accommodation of lithium sheets under confined conditions. Herein, a continuum modeling framework coupling power‐law creep and diffusion is developed to investigate the mechanical behavior of pressed lithium layers of different thickness. It is revealed that lateral shear stress arising from interfacial confinement retards plastic accommodation in lithium layers. This detrimental effect becomes increasingly significant as lithium layers’ thickness *H* decreases or their diameter *D* to thickness *H* ratio (*D*/*H*) increases. For layers of higher *D*/*H*, the stack pressure to realize a constant strain rate is proportional to (*D*/*H*)^(1 + *m*)/*m*^, where *m* is the power‐law creep exponent. Diffusion is beneficial to lithium deformability through reducing interfacial shear stresses and boosting power‐law creep at constant stack pressure. A critical thickness characterizing the dominance of diffusion over creep is theoretically determined and validated through modeling for a wide range of deformation rates. Collectively, these findings advance the fundamental understanding of confined lithium mechanics and provide quantitative guidelines for the structural design and pressure management of ASSLBs.

## Graphical abstract

Plastic accommodation of lithium metal under stack pressure is essential for maintaining Li/SSE interfacial contact. Through continuum modeling, it is revealed that interfacial confinement induces lateral shear stresses and impedes plastic flow. Diffusion mitigates these stresses, enhancing deformability and power‐law creep. A derived critical thickness marks the transition from power‐law creep to diffusion‐dominated behavior, informing ASSLB design.

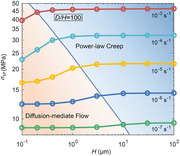

## 1 Introduction

All‐solid‐state lithium‐metal batteries (ASSLBs) hold multiple advantages over traditional Li‐ion batteries in terms of safety, energy density, cycle‐life, and charging‐rate, offering great promise for the future development of energy storage.^[^ ^1^, ^2^, ^3^ ^]^ However, their commercialization is hindered by degradation at the lithium metal–solid‐state electrolyte (SSE) interface during cycling. In particular, lithium stripping often leads to contact loss due to void formation, which increases interfacial impedance and causes localized current concentration.^[^ ^4^, ^5^, ^6^ ^]^ The resulting interfacial contact loss reduces accessible capacity and promotes nonuniform Li deposition, potentially leading to short‐circuiting.^[^ ^7^, ^8^ ^]^ Applying stack pressure is a common strategy to stabilize the Li/SSE interface through mechanically driven creep in lithium metal.^[^ ^9^, ^10^, ^11^ ^]^ It promotes densified and uniform Li deposition^[^ ^12^ ^]^ while suppressing void^[^ ^13^, ^14^ ^]^ and dendrite formation.^[^ ^15^, ^16^ ^]^ However, excessive stack pressure may initiate and propagate cracks in the brittle electrolytes and further facilitate lithium intrusion.^[^ ^17^, ^18^, ^19^ ^]^ Thus, optimizing stack pressure is crucial to ensure both mechanical integrity and electrochemical stability.^[^ ^20^, ^21^, ^22^, ^23^ ^]^

During lithium stripping, the outflux of lithium driven by applied current at the interface must be balanced by creep introduced inflow of lithium to prevent void formation. Achieving this balance is critical for sustaining interfacial contact. In this work, we focus on stack‐pressure‐driven lithium creep, a time‐ and geometry‐dependent deformation process, and elucidate its role in maintaining Li/SSE contact during stripping. Indeed, intensive experimental and theoretical efforts have been devoted to uncovering the underlying deformation mechanisms of lithium metal.^[^ ^24^, ^25^, ^26^, ^27^ ^]^ Several studies reported uniaxial tension experiments on bulk lithium at a wide range of strain rates and temperatures.^[^ ^28^, ^29^, ^30^ ^]^ It has been demonstrated that the plasticity of bulk lithium is primarily governed by shear‐driven dislocation glide which at the macroscopic level follows a power‐law creep behavior. At room temperature, the flow stress is generally limited to 1‐2 MPa. However, nanoindentation,^[^ ^31^, ^32^, ^33^, ^34^ ^]^ micro‐particle,^[^ ^35^, ^36^ ^]^ micro‐, and nanopillar investigations^[^ ^37^, ^38^, ^39^ ^]^ have revealed a pronounced size‐dependent mechanical properties in lithium. As the characteristic length scale decreases from the millimeter to the nanometer range, the yield stress increases by several orders of magnitude, primarily due to the absence in dislocation sources at small scales. It is worth noting that diffusion‐mediated deformation remains active down to the atomic scale, owing to the high homologous temperature of lithium under ambient conditions. Herbert et al.^[^ ^31^, ^32^ ^]^ proposed that, below a certain critical length scale, the dominant deformation mechanism of lithium shifts from the dislocation‐mediated flow to diffusional flow.

Apart from these intrinsic size effects, external mechanical constraints also play an important role in the creep behavior of the lithium metal. It has been reported that the creep rate of lithium foil under compression^[^ ^40^, ^41^ ^]^ is lower by several orders of magnitude than that measured in uniaxial tension of lithium ribbons.^[^ ^28^ ^]^ Moreover, as the aspect ratio increases, the creep rate of lithium foil decreases significantly. This reduction is primarily attributed to the physical confinement imposed by the adjacent layers. Under constant stack pressure and current density, a thinner anode leads to diminished accessible lithium capacity as a result of rapid interfacial degradation.^[^ ^42^ ^]^ This observation is closely related to ASSLBs with layered structures: One may expect that the deformation and stress within lithium layers are strongly influenced by the physical confinement imposed by adjacent layers, particularly when ultrathin lithium foils (*H* < 30 µm) are required to realize high energy densities.^[^ ^43^, ^44^ ^]^ So far, thickness‐dependent creep behavior of lithium layers under confined geometries remains controversial. On one hand, as the anode thickness decreases, the mechanical constraints imposed by the surrounding solid‐state materials become increasingly dominant in governing lithium deformation. On the other hand, thinner lithium layers also exhibit enhanced deformation rates driven by diffusion which may mitigate the stress level. The competitive mechanisms require a deep understanding on thickness‐dependent creep behavior of confined lithium layers in ASSLBs.

In this paper, we employ a theoretical framework to model deviatoric stress driven power‐law creep and hydrostatic pressure driven diffusion within thin lithium layers. The creep behavior of confined lithium layers with varying thicknesses under a constant stack pressure is analyzed. Additionally, we address the inverse problem of examining the required stack pressure to achieve a prescribed strain rate, corresponding to scenarios where batteries discharge at a constant current density. The effects of interfacial confinement are examined, and the relative contributions of power‐law creep and diffusional creep are quantitatively evaluated. Collectively, these findings enhance our understanding of thickness‐dependent creep in lithium layers within all‐solid‐state batteries under stack pressures.

## 2 Results and Discussion

### 2.1 Competitive Deformation Mechanisms

**Figure 1:**
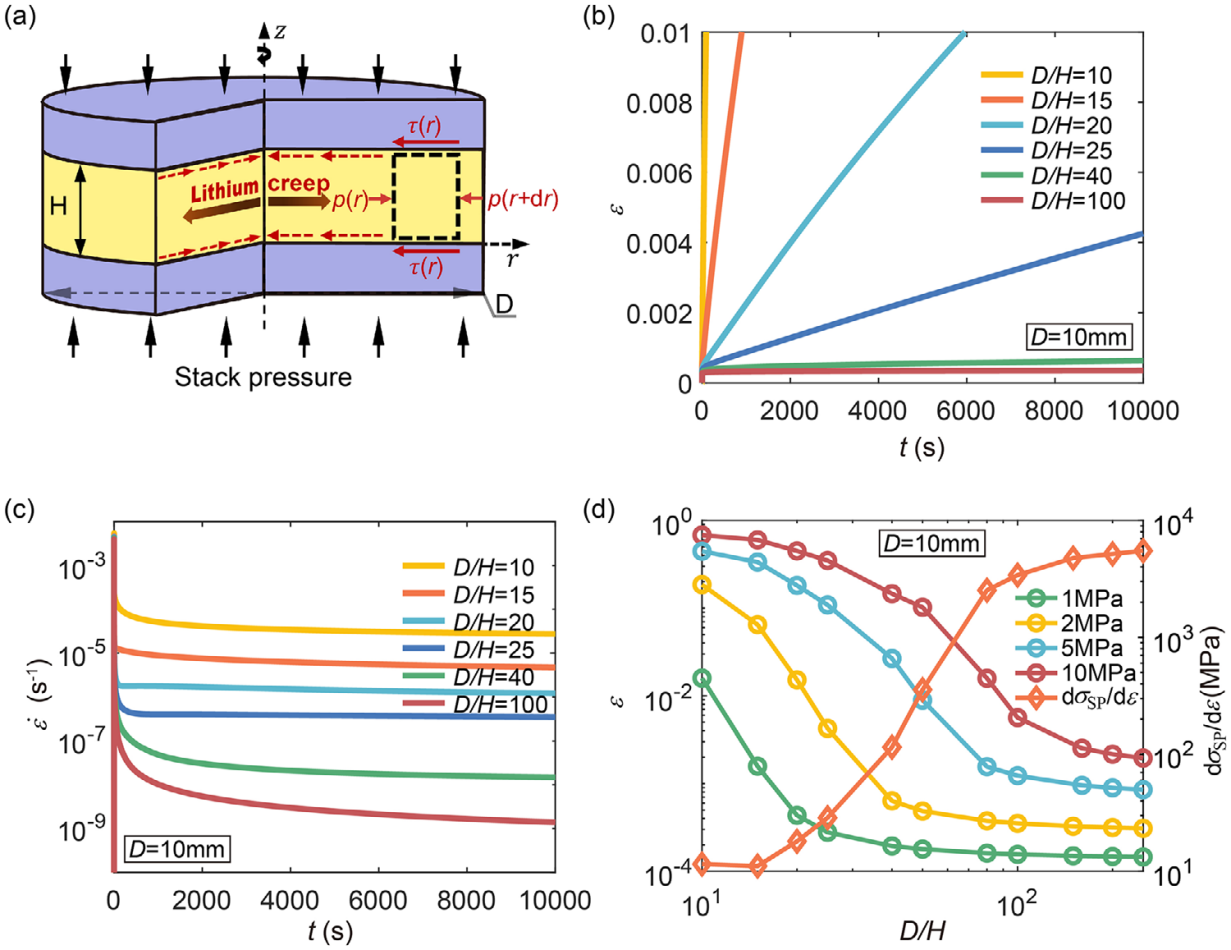
Thickness‐dependent power‐law creep behavior of constrained lithium layers. a) A schematic of a lithium layer (thickness *H* and diameter *D*) under stack pressure, introducing a radial flow of lithium metal and shearing at the Li/SSE interface. b) Nominal creep strain (the ratio of thinned thickness Δ*H* over *H*, i.e., ε = Δ*H*/*H*) versus time curves and c) creep rate ($\dot{\varepsilon }$) versus time curves for lithium layers with varying *H* (*D* = 10 mm and stack pressure σ_SP_ = 2 MPa). (d) Creep strain ε (and dσ_SP_/dε) as a function of *D*/*H* after a 10000 s hold period for σ_SP_ = 1, 2, 5, and 10 MPa. Both ε and $\dot{\varepsilon }$ decrease with decreasing *H* (or increasing *D*/*H*).

As illustrated in **Figure** **1**a, we consider an ASSLB in which a cylindrical lithium layer with thickness *H* and diameter *D* is repeatedly sandwiched. Stack pressure ranging from a few megapascals to tens of megapascals is broadly employed to mitigate void formation at the Li/SSE interface to maintain consistent interfacial contact between different stack layers.^[^ ^5^, ^17^, ^45^ ^]^ Deviatoric stress driven power‐law creep and hydrostatic pressure driven diffusion within thin lithium layers govern the plastic deformation accommodated conformity at the interface.

Building upon the work of Anand and Narayan,^[^ ^46^ ^]^ in which an elastic–viscoplastic theory is employed to model the power‐law creep of lithium, we further incorporate lithium diffusion to provide insights into interface stability at relevant length scales. The continuum theory coupling diffusion and power‐law creep is depicted in the Supporting Information. A no‐slip condition is imposed at the interfaces between lithium and adjacent layers, as supported by experiments showing the full adhesion of lithium to the platens during compression.^[^ ^35^, ^47^ ^]^ The induced mechanical response of the confined lithium layer is studied by finite‐element (FE) simulations using COMSOL Multiphysics. To evaluate the time‐dependent deformation behavior under practical conditions, the creep response of lithium layers with various thicknesses is analyzed under stack pressures of 1, 2, 5 and 10 MPa. Readers are kindly referred to the Supporting Information for detailed parameters and boundary setups in the simulations.

Governing equations associated with the competing deviatoric stress‐controlled creep and hydrostatic pressure driven diffusion are summarized below. The equivalent plastic shear strain‐rate associated with power‐law creep (PC) ${\dot{\bar{\varepsilon }}}^{\mathrm{PC}}$ is expressed as^[^ ^28^ ^]^

(1) $${\dot{\bar{\varepsilon }}}^{\mathrm{PC}}={\dot{\varepsilon }}_{0}{\left(\frac{\bar{\sigma }}{\sigma_{0}}\right)}^{m}$$

where $\bar{\sigma }$ is the von Mises stress, *m* = 6.6 is the power‐law creep exponent, and ${\dot{\varepsilon }}_{0}=7.64\times 10^{-5}\;s$ ^−1^ is the reference creep rate at a reference stress σ_0_ = 0.5 MPa. This value is obtained by evaluating the deformation resistance *s* in Equation S2 (Supporting Information) using its initial value *s*_0_.

A viscosity η* is introduced as^[^ ^48^, ^49^, ^50^ ^]^

(2) $$\eta^{*}=\frac{\bar{\sigma }}{{\dot{\bar{\varepsilon }}}^{\mathrm{PC}}}=\frac{\sigma_{0}}{{\dot{\bar{\varepsilon }}}^{\mathrm{PC}}}{\left(\frac{{\dot{\bar{\varepsilon }}}^{\mathrm{PC}}}{{\dot{\varepsilon }}_{0}}\right)}^{\frac{1}{m}}$$

with which we yield a linear viscous creep of the form

(3) $${\dot{\bar{\varepsilon }}}^{\mathrm{PC}}=\frac{\bar{\sigma }}{\eta^{*}}$$

The volumetric strain rate ${\dot{\varepsilon }}_{V}^{c}$ associated with lithium diffusion is governed by

(4) $${\dot{\varepsilon }}_{V}^{c}=-\Omega_{\mathrm{Li}}\nabla \cdot \vec{j}$$

where Ω_Li_ is the molar volume of lithium, $\vec{j}$ is the diffusional flux of lithium, which is driven by both the lithium concentration gradient and the hydrostatic stress gradient

(5) $$\vec{j}=-D_{s}\nabla c+\frac{D_{s}c\Omega_{\mathrm{Li}}}{RT}\nabla \sigma_{m}$$

where *D*_s_ is the bulk diffusivity, *c* is the molar concentration of lithium, *R* is the gas constant and *T* the temperature, and σ_m_ is the hydrostatic stress. Substituting Equation (5) into Equation (4), the diffusion induced volumetric strain rate becomes

(6) $${\dot{\varepsilon }}_{V}^{c}=D_{s}\Omega_{\mathrm{Li}}\nabla^{2}c-\frac{D_{s}\Omega_{\mathrm{Li}}}{RT}\nabla \left(c\Omega_{\mathrm{Li}}\nabla \sigma_{m}\right)$$

Neglecting the contribution of concentration gradients to mass flux, Equation (6) simplifies to

(7) $${\dot{\varepsilon }}_{V}^{c}=-\frac{D_{s}\Omega_{\mathrm{Li}}}{RT}\nabla^{2}\sigma_{m}$$

Based on Equations (3) and (7), we see a characteristic length scale Λ quantifies the relative rates of diffusional creep over power‐law creep^[^ ^51^, ^52^, ^53^ ^]^

(8) $$\Lambda =\sqrt{\frac{\eta^{*}D_{s}\Omega_{\mathrm{Li}}}{RT}}$$

Using representative values of lithium metals, η* = 3.67 × 10^10^ Pa s and *D*_s_ = 1 × 10^−14^ m^2^ s^−1^ at a strain rate of 10^−5^ s^−1^,^[^ ^49^, ^54^, ^55^ ^]^ we arrive at a characteristic size identifying the dominance between diffusion and power‐law creep of Λ = 1.39 µm. For lithium layers with thickness *H* ≫ Λ, the influence of diffusion on deformation is negligible, and plastic flow is dominated by power‐law creep, as will be detailed in Section 2.2. In contrast, as *H* approaches Λ, diffusion‐mediated flow becomes increasingly important, which will be elaborated in Section 2.3.

### 2.2 Power‐Law Creep Dominance

In this section, we investigate the plastic deformation of lithium layers with thickness *H* ranging from 40 to 1000 µm, while keeping the diameter *D* = 10 mm constant. The resultant aspect ratio *D*/*H* varies from 10 to 250, serving as a key geometric parameter that characterizes the degree of interfacial confinement. Although these *D*/*H* ratios are relatively large, they are representative of the actual geometry of lithium layers in stacked solid‐state battery configurations, where the lateral dimension of the electrode is typically much greater than its thickness. Figure 1b shows the time‐dependent creep strain curves for lithium layers subjected to a stack pressure of 2 MPa with several *D*/*H*. The thinner the lithium layer and the higher its aspect ratio, the slower it deforms plastically. From the strain rate versus time curves shown in Figure 1c, we find that the creep rates for thin lithium layers under stack pressure, when geometrically constrained as shown in Figure 1a, are several orders of magnitude lower than those from uniaxial tension tests—lithium ribbons under uniaxial tension about 0.5 MPa may creep at a rate of 4 × 10^−5^s^−1^.^[^ ^28^ ^]^ It is a manifestation of the significance of factoring in geometry issues when designing ASSLBs. The total creep strain under a constant stress holding for 10 000 s as a function of *D*/*H* is presented in Figure 1d. At those stack pressures explored, increasing *D*/*H* leads to a reduction in the total creep strain, suggesting lower deformability in thinner lithium layers: thinner lithium layers require higher stack pressures to suppress voids.

**Figure 2:**
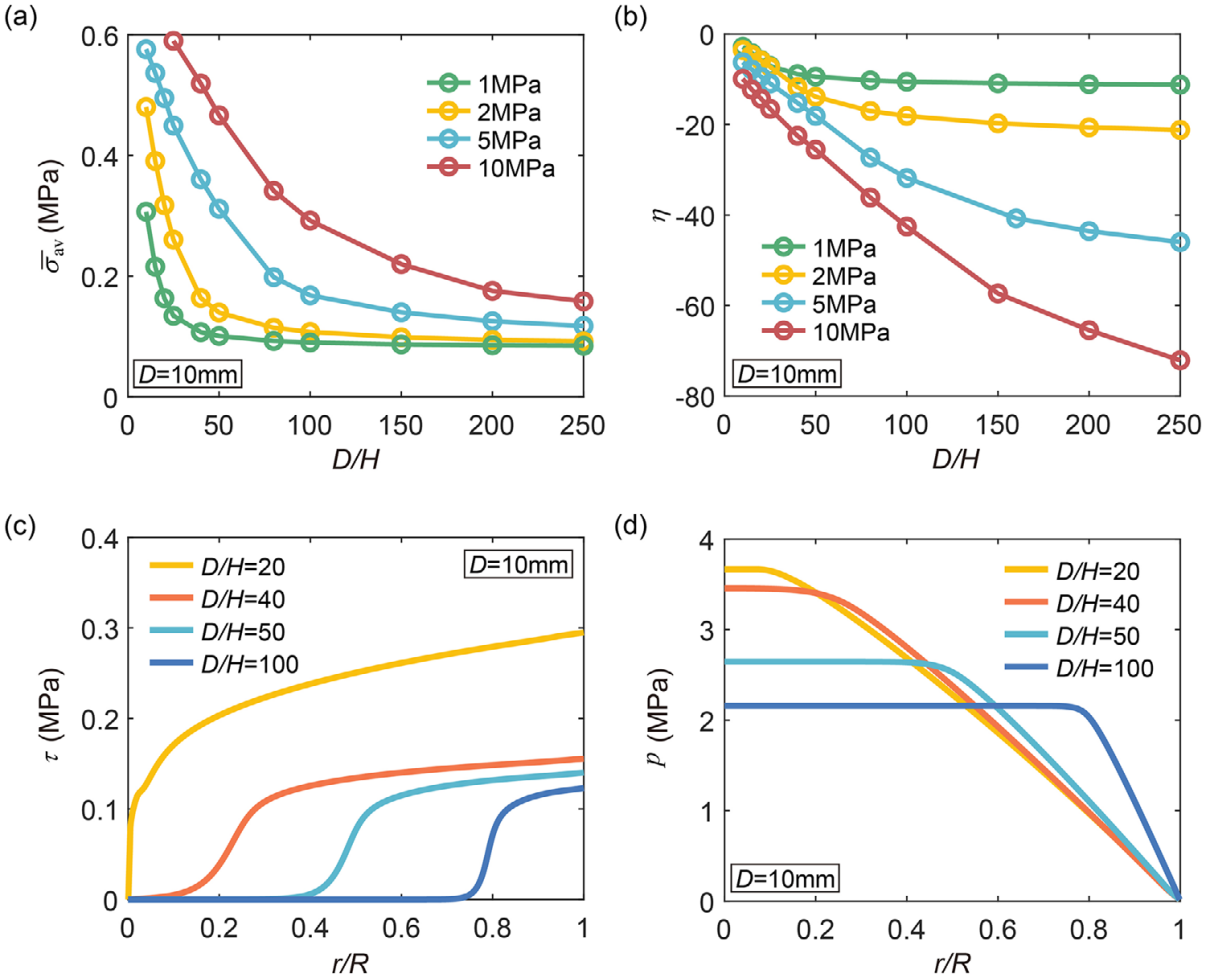
Macroscopic stress state of lithium layers under constant stack pressure. a) Average von Mises stress ${\bar{\sigma }}_{\mathrm{av}}$ and b) average stress triaxiality η curves as a function of *D*/*H* for lithium layers under different stack pressures. c) Distribution of shear stress τ and d) distribution of pressure *p* at the interface under a 2 MPa stack pressure. Here *r* denotes the radial position along the interface and *R* = *D*/2 is the radius of the battery. Interfacial shear stress leads to the buildup of hydrostatic stress and a reduction in von Mises stress.

The thickness‐dependent power‐law creep behavior of lithium layers (Figure 1) is attributed to transverse adhesive forces at the interface during compression. A multiaxial stress state develops within the thin lithium layer, albeit the uniaxial nature of the applied stack pressure. **Figure** **2**a shows the average von Mises stress (${\bar{\sigma }}_{\mathrm{av}}$) within lithium layers as a function of *D*/*H* under different stack pressure. Notably, while the stack pressures are 1, 2, 5 and 10 MPa, the von Mises stress for thin lithium layers of high aspect ratio (*D*/*H*> 200) is only about 0.085, 0.09, 0.12, and 0.18 MPa, respectively. As *D*/*H* increases from 10 to 100, the von Mises stress decreases by approximately 4.5 times when taking a stack pressure of 2 MPa as an example. Given the exponent of the creep law in Equation (1) is about 6.6,^[^ ^28^ ^]^ such a reduction in equivalent stress results in nearly four orders‐of‐magnitude decrease in creep rate, which agrees well with the thickness‐dependent strain rate shown in Figure 1c.

The variation in the von Mises stress (and consequently the creep rate) with *D*/*H* is a direct resultant from the interfacial constraints. The degree of these constraints can be quantified by stress triaxiality η, defined as the ratio of hydrostatic stress (σ_m_) to the von Mises stress ($\bar{\sigma }$)

(9) $$\eta =\frac{\sigma_{m}}{\bar{\sigma }}$$

For uniaxial compression, η = −0.33, whereas for triaxial equivalent compression, η tends toward negative infinity. Figure 2b shows the average triaxiality factor within lithium layers as a function of *D*/*H* under different stack pressures. As layer thickness drops, the triaxiality factor (negative in compression) decreases rapidly. This triaxiality evolution highlights the impact of reducing layer thickness on altering the macroscopic stress state of lithium, and hence the creep rate.

In order to evaluate how reduced layer thickness amplifies interfacial confinement effects, we examine the distributions of shear stress τ and pressure *p* at the interface under a stack pressure of 2 MPa. As shown in Figure 2c, for thinner layers (and hence higher *D*/*H*), the interfacial shear stress is lower. To understand this counterintuitive result, we refer to the free‐body diagram of the constrained lithium layer as shown in Figure 1a. Given that *H* ≪ *D*, the problem can be simplified to a 1D axisymmetric case, where the local stresses depend only on the radial position *r*. Force balance in the radial direction of an infinitesimal element near the layer edge (see Figure 1a) requires the shear stress τ(*r*) and the pressure *p*(*r*) to follow

(10) $$\frac{dp\left(r\right)}{dr}=-\frac{2\tau \left(r\right)}{H}\;$$

Equation (10) indicates that the shear stress governs the pressure build up in thin layers. Integrating Equation (10), we have

(11) $$p\left(r\right)=\frac{1}{H}\int_{r}^{R}2\tau \left(r\right)\;dr$$

For the geometry shown in Figure 1a, force balance in the normal direction of the Li/SSE interface requires the average normal stress (or the average pressure) approximate to the stack pressure σ_SP_, regardless of the aspect ratio *D*/*H*. Therefore, to maintain the same average pressure as the layer becomes thinner (i.e., decreasing *H*), the shear stress τ(*r*) must correspondingly decrease. Interestingly, although the shear stress diminishes with decreasing *H*, the pressure rises more steeply near the layer edges in thinner layers (*r*/*R* = 1 in Figure 2d). Combined with Equation (11), it can be concluded that the geometric factors associated with reduced *H* or increased *D*/*H* dominate the buildup of pressure at the interface. At the central region of the interface (*r*/*R* = 0 in Figure 2c,d), the shear stress approaches to zero and the pressure remain constant, yielding a purely hydrostatic stress state.

### 2.3 Diffusion Dominance

**Figure 3:**
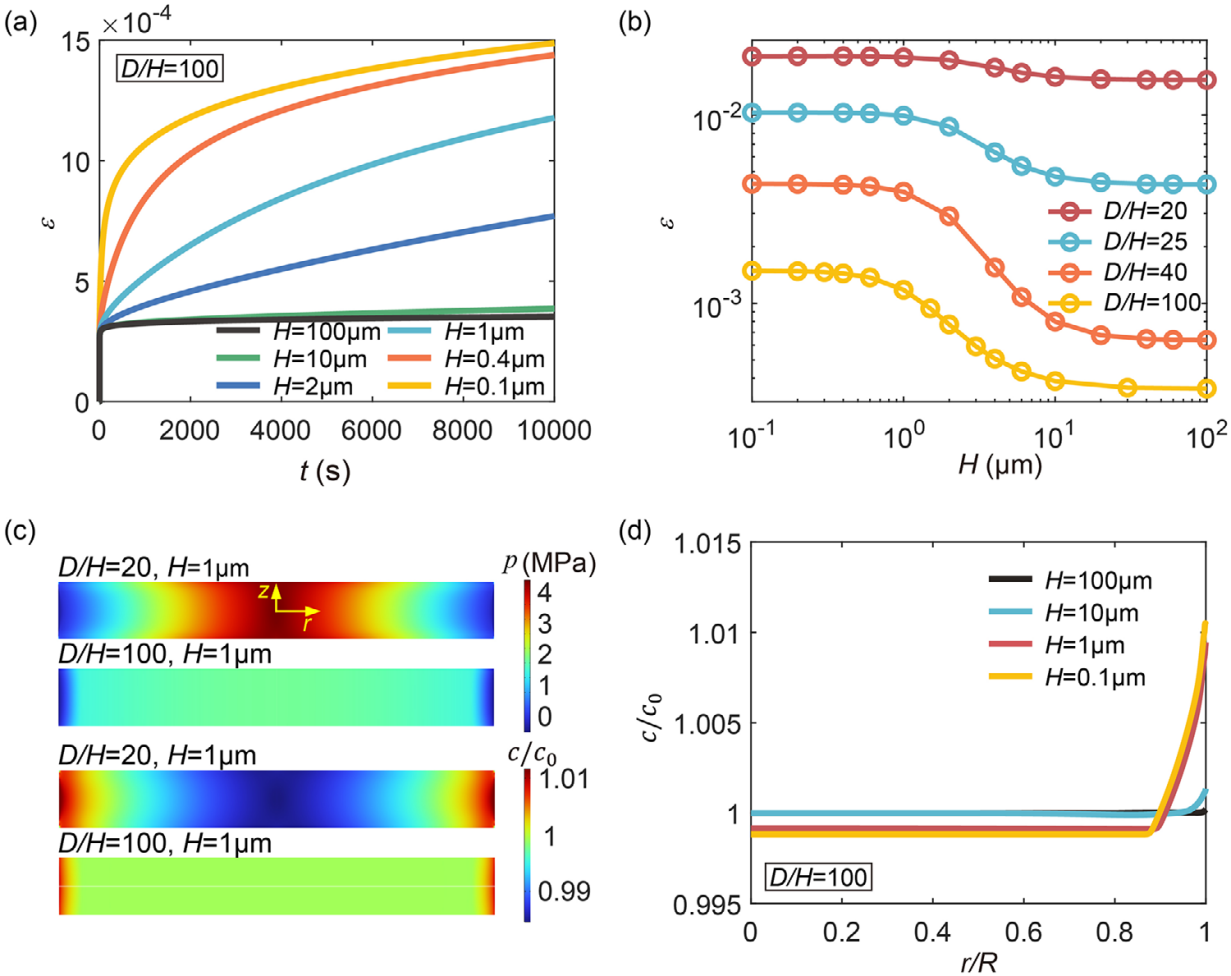
Diffusion‐mediated flow in thin lithium layers. a) Creep strain versus time curves for lithium layers with a fixed ratio *D*/*H* = 100 and varying *H*, under a stack pressure of 2 MPa. b) Creep strain after a 10 000 s dwell under a stack pressure of 2 MPa, as a function of *H*, for several fixed *D*/*H* ratios. c) Pressure distribution and normalized lithium concentration *c*/*c*_0_ profiles in lithium layers with *H* = 1 µm and *D*/*H* = 20 and 100 at the end of stress loading. Here *c* is the molar concentration of lithium, and *c*_0_ is the equilibrium concentration under stress‐free state. d) Normalized lithium concentration *c*/*c*_0_ along the interface in lithium layers with *D*/*H* = 100 and various *H*.

In relatively thick lithium layers, the diffusion is of bulk nature. However, as the layer thickness *H* becomes comparable to the characteristic length scale Λ, diffusion through the channels of the interfaces takes control and diffusion mediated deformation would become dominant. To explore the role of diffusion in the deformation behavior of confined thin lithium layers, we consider cases with a fixed *D*/*H* and systematically reduce the layer thickness from 100 to 0.1 µm. **Figure** **3**a presents the time‐dependent creep strain under a stack pressure of 2 MPa for lithium layers with *D*/*H* = 100 and various *H*. It is observed that the creep strain increases as *H* decreases, suggesting an enhanced contribution from diffusion‐mediated flow in thinner layers. In Figure 3b, the total creep strain after a constant stress hold of 10 000 s is plotted as a function of *H* for several *D*/*H*. In general, for the layers with *H* > 10 µm, the contribution of diffusion to deformation is negligible. A pronounced increase in total creep strain is observed as *H* decreases from 10 to 1 µm, indicating a transition in the dominant deformation mechanism. However, for the ultrathin layers with *H* < 1 µm, the total creep strain plateaus and no longer increases with further reduction in thickness, suggesting that diffusion has reached a saturation‐dominated regime.

As described in Section 2.2, a pressure gradient may develop within the lithium layer during constrained compression due to the interfacial shear stress. This gradient drives lithium diffusion from regions of higher pressure to those of lower pressure. Figure 3c illustrates the pressure distribution and the normalized lithium concentration profile in lithium layers with *H* = 1 µm and *D*/*H* = 20 and 100 at the end of loading. It is shown that pressure concentrates near the center of the layer and decreases toward the edges. Driven by this pressure gradient, lithium diffuses outward from the layer interior toward the edges. Figure 3d presents the normalized lithium concentration *c*/*c*_0_ along the interface for cases with *D*/*H* = 100 and various *H*. For layers with *H* > 10 µm, the variation in *c*/*c*_0_ is small, indicating limited diffusion. In contrast, when *H* ≈ 1 µm, lithium tends to accumulate near the layer's edges, underscoring the reinforced diffusion effect on lithium distribution.

**Figure 4:**
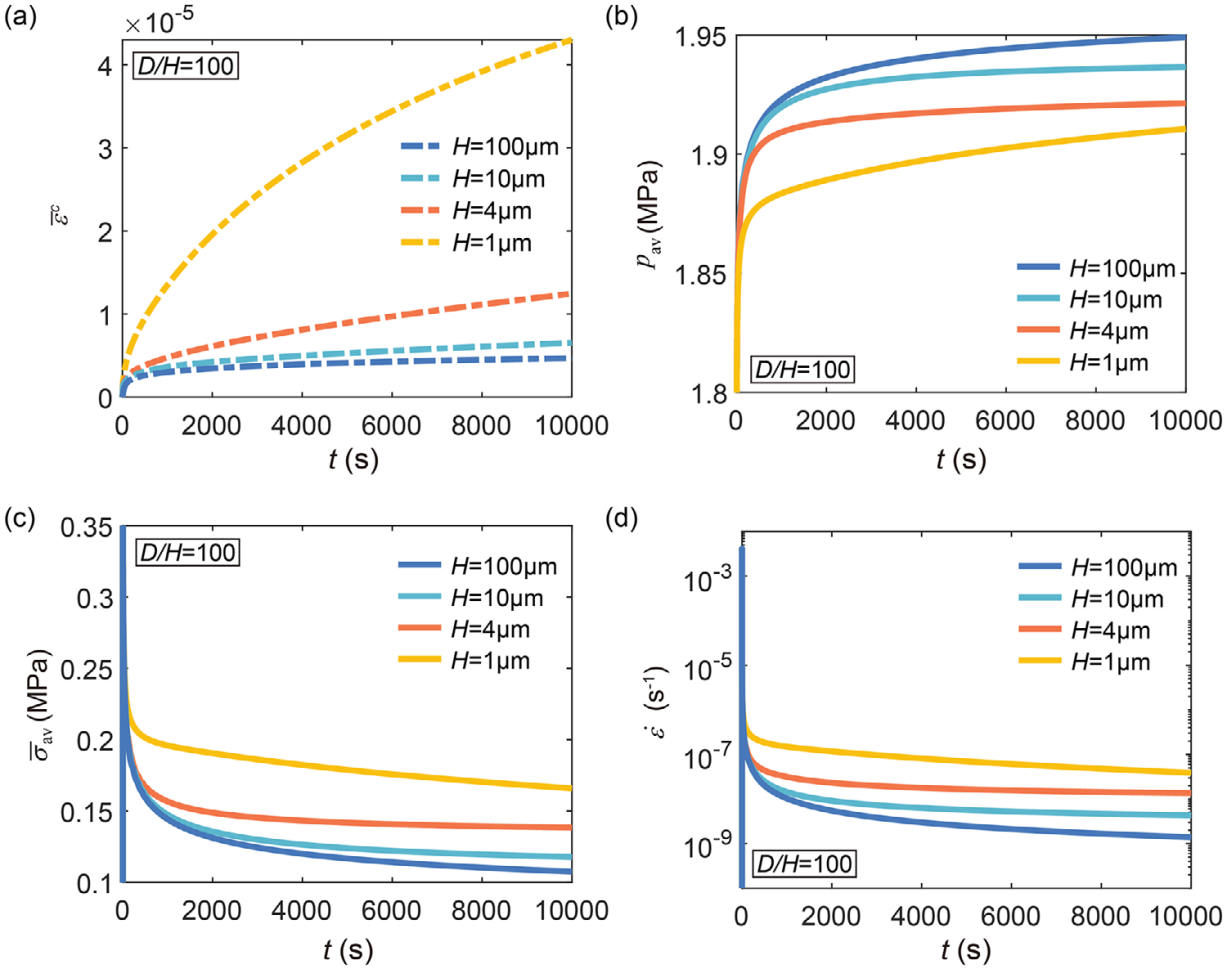
Diffusion‐mediated creep mechanism for various *H*. a) Time evolution of the overall creep strain induced by lithium redistribution ${\bar{\varepsilon }}^{c}$ for lithium layers with a fixed *D*/*H* = 100. b) Time evolution of the average hydrostatic pressure *p*_av_ and c) time evolution of the average von Mises stress ${\bar{\sigma }}_{\mathrm{av}}$ in lithium layers with a fixed *D*/*H* = 100. d) Creep rate versus time curves for lithium layers with a fixed *D*/*H* = 100. As *H* approaches Λ (Equation 8), diffusion becomes more effective in mitigating pressure buildup, resulting in higher von Mises stress and enhanced power‐law creep.

Although mass transport by atomic diffusion induces localized volumetric strains, the overall creep strain ${\bar{\varepsilon }}^{c}$ due to lithium redistribution remains negligible (see ${\bar{\varepsilon }}^{c}$ in **Figure** **4**a compared to ε in Figure 3a). Nevertheless, lithium redistribution modifies the evolution of internal pressure, thereby influencing the local stress state and the overall creep behavior. Figure 4b,c shows the time evolution of the average hydrostatic pressure *p*_av_ and average von Mises stress ${\bar{\sigma }}_{\mathrm{av}}$, respectively, for lithium layers with a fixed *D*/*H* = 100 and different *H*. As loading proceeds, hydrostatic pressure gradually increases, while the average von Mises stress decreases. Figure 4d shows the corresponding time‐dependent creep rate, which declines with time in accordance with the von Mises stress reduction in Figure 4c. By comparing the time‐dependent pressure evolution in lithium layers with different *H* (Figure 4b), we find that pressure builds up more rapidly in thicker layers. In contrast, in thinner layers, diffusion more effectively alleviates pressure accumulation, leading to higher von Mises stress and enhanced power‐law creep (Figure 4c,d).

### 2.4 Compression under Constant Strain Sates

To this point, we have investigated the thickness‐dependent creep behavior of constrained lithium layers imposed with constant stack pressure. In engineering practice, we are concerned with the inverse issue that the batteries are discharging at a constant current density. The flux of lithium vacancies into the metal driven by the applied current density must be balanced by mechanical deformation of the lithium to ensure interfacial coherence at the Li/SSE interface during stripping. In this context, the current density imposes an effective deformation rate at the interface, representing the rate of volumetric change required to compensate for the electrochemical removal of Li atoms. This effective deformation rate has been used as a first‐order approximation to determine the stack pressure needed to sustain a certain stripping current density.^[^ ^13^, ^56^, ^57^ ^]^

Accordingly, the average effective deformation rate corresponding to a given current density *i* can be expressed as^[^ ^13^, ^56^, ^57^ ^]^

(12) $$\dot{\varepsilon }=\frac{i}{F}\frac{\Omega_{\mathrm{Li}}}{H}$$

where *F* is the Faraday's constant. For a typical current density of *i* = 1 mA cm^−2^ and layer thickness of *H* = 100 µm, the resulting effective deformation rate is $\dot{\varepsilon }=1.35\times 10^{-5}s^{-1}\;$. In the following analysis, we use strain‐controlled boundary condition to explore the evolution of stack pressure.

**Figure 5:**
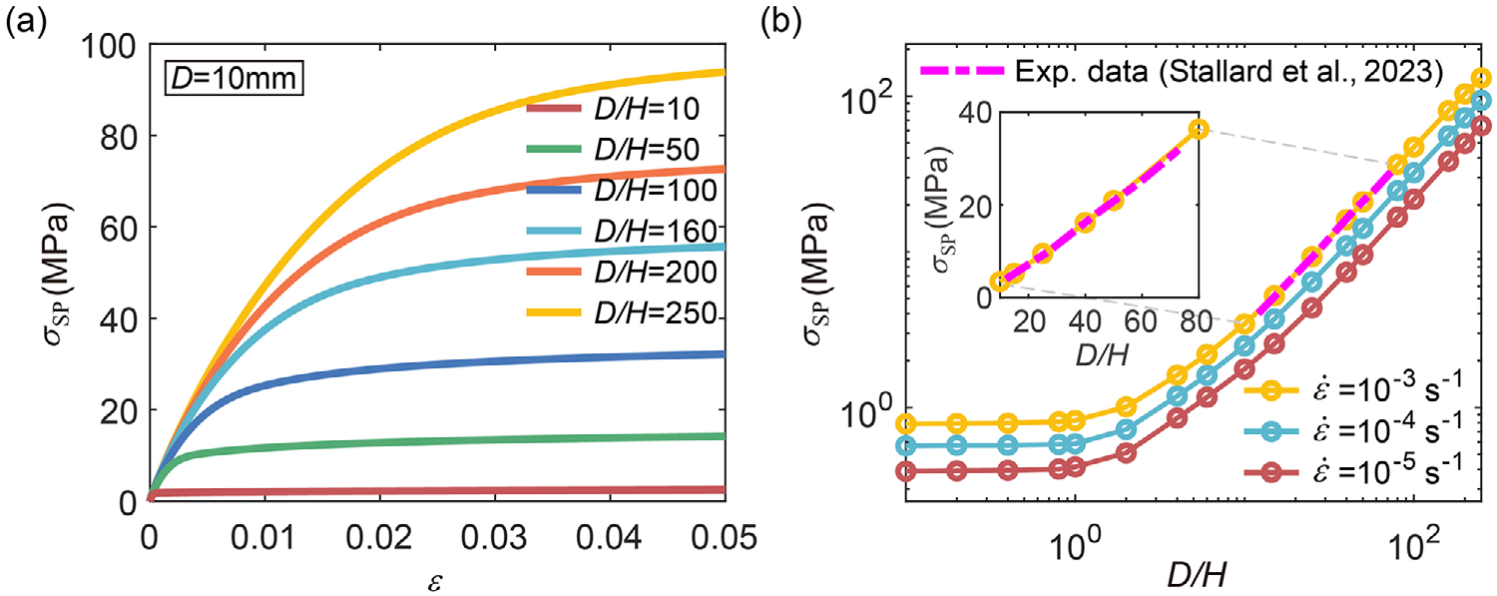
Constrained compression of lithium layers under constant strain rates. a) Stress–strain curves for lithium layers with fixed diameter *D* = 10 mm and various *D*/*H* at a strain rate of 10^−4^ s^−1^. b) Stack pressure σ_SP_ at a strain of ε = 0.05 as a function of aspect ratio (*D*/*H*) under applied strain rates of 10^−3^, 10^−4^, and 10^−5^ s^−1^. For thin layers (*D*/*H* > 10), σ_SP_ is proportional to (*D*/*H*)^(1 + *m*)/*m*^. Note the experimental results^[^ ^35^ ^]^ fit well with σ_SP_ at the strain rate of 10^−3^ s^−1^.

Following the approach in Sections 2.2 and 2.3, we first consider relatively thick lithium layers where power‐law creep dominates, and then examine thinner layers where diffusion becomes increasingly significant. Consistent with Section 2.2, the lithium layer diameter is fixed at *D* = 10 mm, while the thickness *H* (or equivalently, *D*/*H*) varies. **Figure** **5**a shows stress–strain responses for lithium layers with several *D*/*H* under an applied strain rate of 10^−4^ s^−1^. The induced stress, i.e., the required stack pressure σ_SP_, increases progressively with deformation due to the accumulation of internal stress within the lithium layer. For thick layers with low aspect ratios, σ_SP_ remains limited to a few MPa. In contrast, for thinner layers with higher *D*/*H* or smaller *H*, σ_SP_ can exceed hundreds of MPa. Figure 5b presents the stack pressure σ_SP_ at a strain of ε = 0.05 as a function of *D*/*H*, under applied strain rates of 10^−3^, 10^−4^ and 10^−5^ s^−1^, respectively. For the thick layers with *D*/*H* < 1, σ_SP_ at different strain rates are close to those of unconstrained uniaxial compression. However, as layer thickness decreases or aspect ratio increases, σ_SP_ increases significantly. With regard to the thin layers of *D*/*H* > 10, σ_SP_ scales with (*D*/*H*)^(1 + *m*)/*m*^. This scaling relationship between σ_SP_ and *D*/*H* is consistent with the approximate analytic solution of Cheung and Cebon^[^ ^58^ ^]^ for forging of a thin power‐law creeping film. Figure 5b also includes experimental data from Stallard et al.,^[^ ^35^ ^]^ where a lithium sphere with an initial diameter of 1260 µm was compressed at a strain rate of 10^−3 ^s^−1^. During compression, the height of the lithium sphere decreased while its diameter increased, accompanied by a steep rise in the average pressure. Our calculations agree well with these experimental observations. It is noted that the compression data of Stallard et al.^[^ ^35^ ^]^ were obtained from cast lithium. Their macroscopic uniaxial tensile response is closed to that of rolled Li foils tested by LePage et al.,^[^ ^28^ ^]^ which exhibited no significant in‐plane texture and isotropic tensile behavior. Since the constitutive parameters used in our model were calibrated using the isotropic response of rolled foils,^[^ ^28^ ^]^ the stress–strain data of Stallard et al.^[^ ^35^ ^]^ provide a physically consistent validation of the model. The potential influence of grain size and texture on the creep behavior of Li foils may be explored in future work. Notably, the stress levels in the experimental data differ significantly (Figure S1b, Supporting Information vs Figure 5b): the resultant stress is below 1 MPa for Li ribbons in uniaxial tension at a strain rate of 3 × 10^−3 ^s^−1^, whereas it exceeds 20 MPa for thin Li layers with large aspect ratios (*D*/*H*  >  50) under compression at 1 × 10^−3 ^s^−1^. Our model is capable of well capturing the role of external constraint in elevating the average pressure at large aspect ratios.

**Figure 6:**
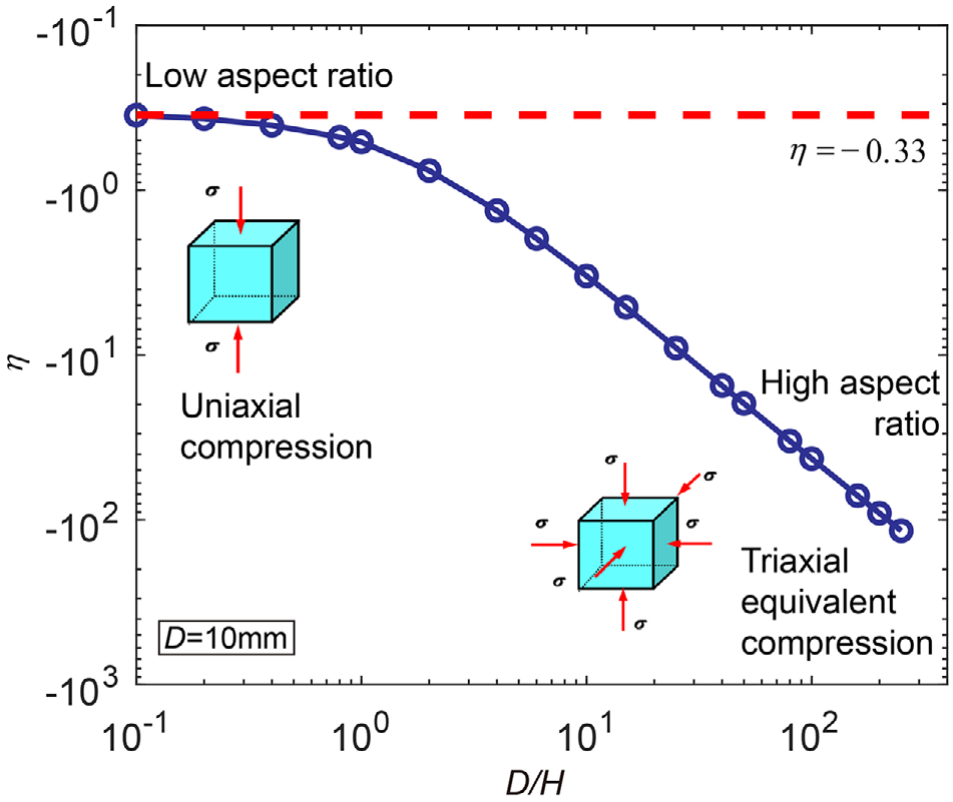
Average stress triaxiality η as a function of aspect ratio (*D*/*H*) for lithium layers subjected to a strain‐controlled boundary condition at the strain rate of 10^−4^ s^−1^.

**Figure** **6** further illustrates the average stress triaxiality of the lithium layer as a function of *D*/*H*. For thick lithium with low aspect ratios (*D*/*H* < 0.1), the stress triaxiality remains constant at ‐0.33, indicating the mechanical state in thick lithium is dominated by uniaxial compression with negligible influence from interfacial confinement. However, as *D*/*H* increases, the stress triaxiality drops sharply, indicating a transition to a highly triaxial stress state dominated by interfacial confinement.

**Figure 7:**
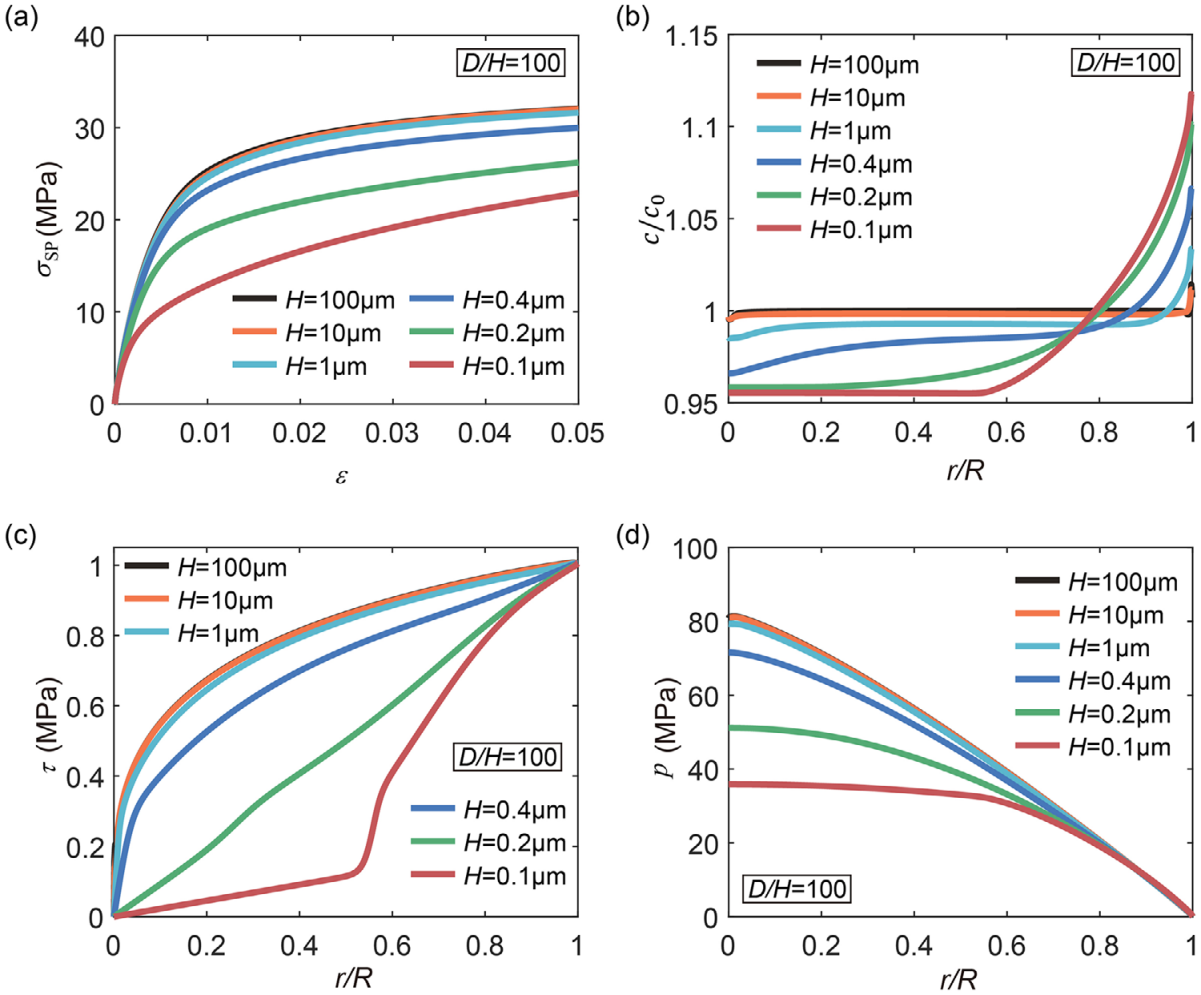
Diffusion‐based stress relaxation. a) Stress–strain curves for lithium layers with a fixed aspect ratio *D*/*H* = 100 and various *H* at a strain rate of 10^−4^ s^−1^. b) Normalized lithium concentration *c*/*c*_0_ distribution, c) shear stress distribution and d) pressure distribution along the interface for lithium layers with different *H* at a strain rate of 10^−4^ s^−1^. b–d) *r* denotes the radial position along the interface, with *r* = 0 corresponds to the center of the layer.

Next, we examine thin lithium layers where diffusion become more pronounced, considering samples with a fixed *D*/*H* = 100 and varying thickness *H*. **Figure** **7**a displays the stress–strain responses of lithium layers with thicknesses ranging from 100 to 0.1μm, subjected to a constant strain rate of 10^−4^ s^−1^. It is evident that thinner layers exhibit slower stress accumulation and lower stress levels. Figure 7b presents the corresponding profiles of normalized lithium concentration *c*/*c*_0_ along the interface at a strain of ε  =  0.05. For relatively thick layers (*H* > 1 µm), the variation in *c*/*c*_0_ with radial position *r* is minimal, suggesting limited diffusion‐induced mass transport. In contrast, ultrathin layers (*H* < 1 µm) exhibit pronounced spatial gradients in *c*/*c*_0_, underscoring the increasingly active role of diffusion in redistributing lithium as *H* decreases. To further elucidate the mechanism of diffusion‐driven stress relaxation, we examine the stress distribution along the interface. Figure 7c,d illustrates the radial distributions of interfacial shear stress and pressure at the end of loading for lithium layers with varying *H*. As *H* decreases, the interfacial shear stress diminishes significantly. This is accompanied by a corresponding drop in pressure, in accordance with Equation (11). These results highlight that in sufficiently thin lithium layers, diffusion effectively relieves interfacial shear stress and reduces the overall stress levels.

**Figure 8:**
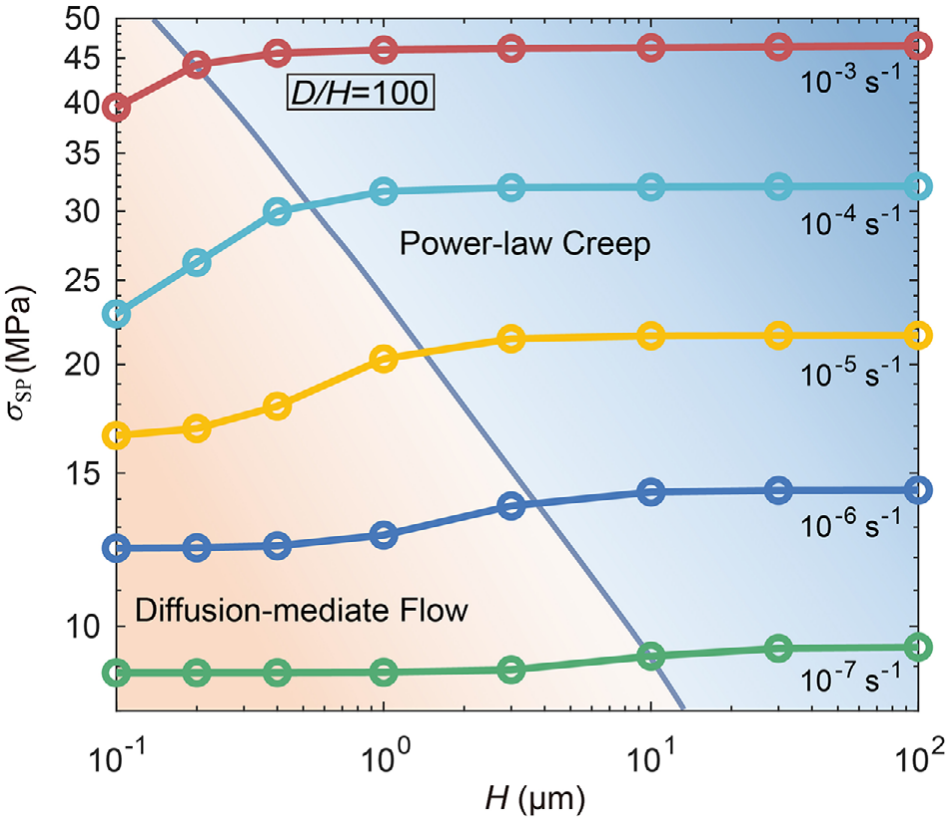
Deformation mechanism maps for lithium layers of all‐solid‐state batteries, showing how deformation regimes evolve with layer thickness and applied strain rate. Solid curves indicate the dependence of stack pressure σ_SP_ on layer thickness *H* at a strain of ε = 0.05 under different strain rates. The map delineates the transition between diffusion‐mediated and power‐law creep–dominated regimes. The transition boundary is determined based on Equation (8).

The above results suggest the existence of a critical length scale below which diffusion‐mediated processes become increasingly significant in determining the mechanical response of lithium. To elucidate this transition, **Figure** **8** presents the deformation mechanism maps for constrained lithium layers with fixed *D*/*H* = 100. The solid curves depict how the stack pressure at a strain of ε = 0.05 varies with layer thickness under different prescribed strain rates. In the regime of large layer thickness (H>10μm), σ_SP_ remains largely insensitive to thickness, indicating that the deformation is dominated by dislocation‐mediated power‐law creep and that diffusion plays a negligible role in stress relaxation. However, as the layer becomes thinner, a pronounced decrease in σ_SP_ is observed, reflecting the enhanced contribution of diffusion to stress relaxation. This behavior delineates two distinct deformation regimes: a power‐law creep‐dominated regime at larger thicknesses and higher stress levels, and a diffusion‐mediated regime at reduced thicknesses and lower stresses. The transition boundary between these regimes shifts toward smaller thicknesses with increasing strain rate. This trend reflects the strain‐rate dependence of the characteristic length scale Λ associated with power‐law creep, as defined by Equation (8), and is consistent with the nanoindentation results of Herbert et al.,^[^ ^31^ ^]^ which identified a characteristic length of ≈400 nm at an indentation strain rate of 0.006 s^−1^ and a reduction of this length with increasing strain rate.

## 3 Conclusion

This work establishes mechanistic insights into the thickness‐dependent creep behavior of confined lithium layers. While previous studies mostly used Li foils of 100 −750 µm to evaluate the effect of stack pressure, practical ASSLBs will require ultrathin Li anodes (*H* < 30 µm) to realize their full energy density potential.^[^ ^43^, ^44^ ^]^ Such thickness reduction severely limits the anode deformability needed to maintain interfacial contact under pressure. Here, we systematically investigate how confinement and geometry govern the creep response of lithium layers and determine the stack pressure required for stable operation in all‐solid‐state lithium‐metal batteries.

Through our investigation, we reveal that a multiaxial stress state develops within the lithium layers due to the lateral shear forces generated at the interface. Consequently, under constant stack pressure, both the von Mises stress and the associated creep rate are substantially reduced compared to unconstrained compression. This confinement effect becomes increasingly significant as the layer thickness *H* decreases or the aspect ratio (*D*/*H*) increases. Under strain‐controlled loading, remarkably higher stack pressures are required in thinner lithium layers. The stack pressure required to maintain a constant strain rate scales as (*D*/*H*)^(1 + *m*)/*m*^, in which *m* is the power‐law creep exponent for bulk lithium. The buildup of pressure is directly linked to the interfacial shear stress. These results underscore interfacial shear stress as the critical factor governing both the suppression of creep deformation under constant pressure and the increased stack pressure demands under strain‐controlled loading in thinner lithium layers. Under constant stack pressure, diffusion alleviates hydrostatic stress accumulation, leading to a higher von Mises stress, and thus promoting power‐law creep. Under strain‐controlled loading, diffusion reduces the interfacial shear stress, resulting in lower overall stress levels within thin lithium layers. We further identify a critical thickness Λ (Equation 8), below which diffusional processes significantly influence the mechanical response. Within the examined strain rates, this critical thickness falls between several micrometers and sub‐micrometer scales, and decreases with increasing strain rate.

Overall, we adopt a continuum modeling framework to investigate the macroscopic deformation behavior of confined lithium layers, without delving into microscopic details such as porosity, grain size, or dislocation topology. Future investigations are needed to coupling the microscopic and macroscopic effects in theoretical and experimental analyses. While this present approach simplifies the inherently complex problem, e.g., the thickness dependent flow strength is neglected here, it helps establishing a baseline mechanistic understanding of lithium deformation with geometry confinement. Our findings highlight how the interplay among dislocation‐mediated plasticity, atomic diffusion, interfacial adhesion, and geometric confinement governs the overall stress state and creep behavior within lithium layers. From a practical perspective, this framework provides quantitative guidance for determining stack pressure across different lithium geometries and operating conditions. Figure S2 in the Supporting Information presents examples of the relationship between optimal stack pressure and lithium layer thickness at different current densities. The insights gained from these analyses provide valuable guidance for structural design and pressure management in ASSLBs, with the aim of enhancing both mechanical integrity and electrochemical performance in practical applications.

## Conflict of Interest

The authors declare no conflict of interest.

## Supporting information



Supporting Information

## Data Availability

The data that support the findings of this study are available from the corresponding author upon reasonable request.
