# Supplementary material for: Thickness‐Dependent Creep in Lithium Layers of All‐Solid‐State Batteries under Stack Pressures
Source: Adv Sci (Weinh). 2025 Nov 16;13(6):e17361. doi: 10.1002/advs.202517361 (PMC12866736; doi:10.1002/advs.202517361)
Supplement: Supplementary file 1 — Supporting Information [file ADVS-13-e17361-s001.docx]

**Supporting Information**

**Thickness-dependent creep in lithium layers of all-solid-state batteries under stack pressures**

Chuangchuang Duan^1,2^, Yiming Feng^1,2^, Tianliang Lin^1,2*^, Ruifang Ye^1,2^, Mingqiang Li^3,4^, Jici Wen^3,4^, Chunguang Chen^3,4*^, and Yujie Wei^3,4*^

^1^College of Mechanical Engineering and Automation, Huaqiao University, Xiamen 361021, China

^2^Fujian Key Laboratory of Green Intelligent Drive and Transmission for Mobile Machinery, Huaqiao University, Xiamen 361021, China

^3^LNM, Institute of Mechanics, Chinese Academy of Sciences, Beijing 100190, China

^4^School of Engineering Sciences, University of Chinese Academy of Sciences, Beijing 100049, China

**Correspondence*: ltl@hqu.edu.cn, chenchunguang@imech.ac.cn, yujie_wei@lnm.imech.ac.cn

**Model description**

The axisymmetric compression problems are solved by finite element (FE) method. Figure S1a illustrates the model used in finite element (FE) calculations. The axisymmetric and symmetric boundary conditions are employed on the left and bottom edges, respectively. A constant velocity or pressure is imposed at the top edge of the model. The adjacent layers are treated as rigid materials. Full adhesion is assumed at the interfaces between lithium and adjacent layers, which means zero tangential velocity along the interface $z=H/2$. Under applied stack pressure, lithium metal exhibits deformation through multiple mechanisms, including elastic deformation, diffusional creep and power-law creep.


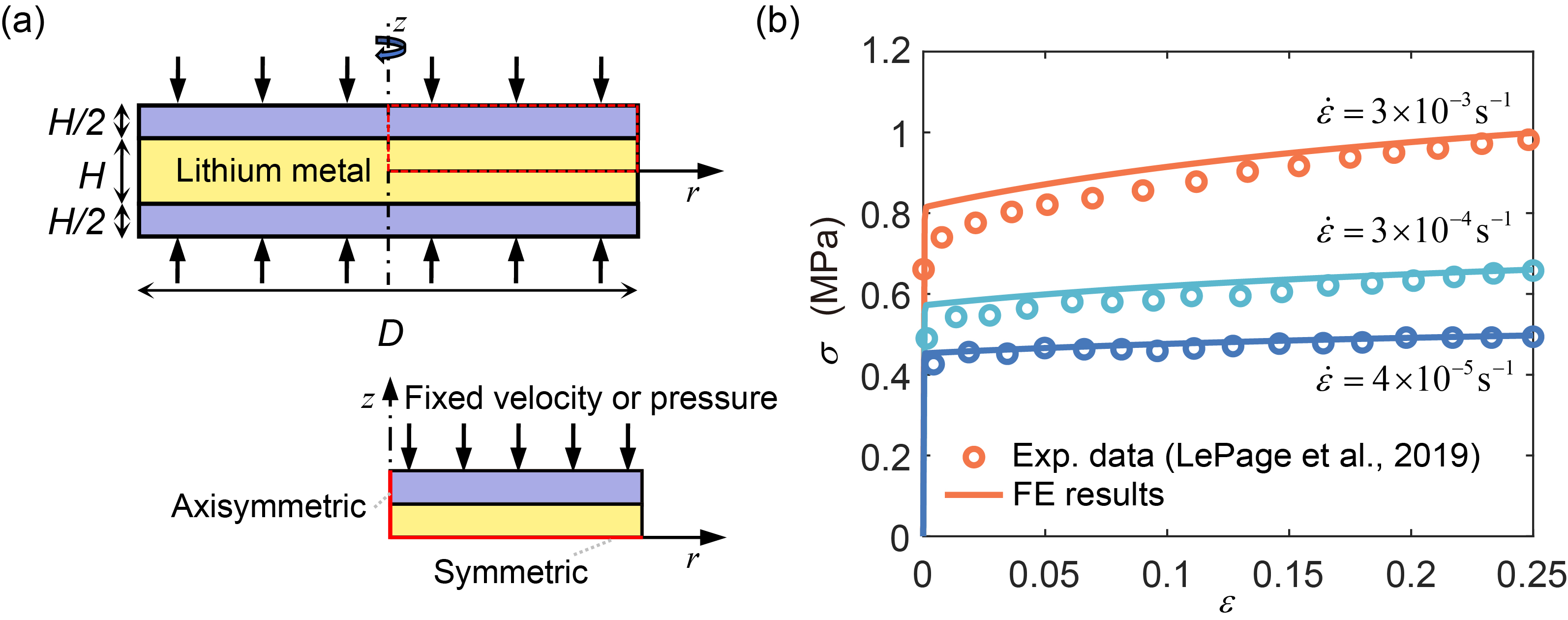


**Figure S1.** (**a**) Schematic of an axisymmetric model used in the simulations. (**b**) Comparison of stress-strain curves for unconstrained uniaxial tension at different strain rates, between experimental data^[5]^ (symbols) and FE simulation results (lines) using parameters listed in Table S2.

***Deformation model for Li metal***

The total strain $\boldsymbol{\varepsilon}$ can be decomposed into elastic, viscoplastic, and chemical components as^[1-3]^

$\boldsymbol{\varepsilon}=\boldsymbol{\varepsilon}^{e}+\boldsymbol{\varepsilon}^{\mathrm{PC}}+\boldsymbol{\varepsilon}^{c}.$ (S1)

The elastic strain $\boldsymbol{\varepsilon}^{e}$ is related to the stress tensor $\boldsymbol{\sigma}$ by Hooke’s law

$\boldsymbol{\varepsilon}^{e}=\frac{1+v_{Li}}{E_{Li}}\boldsymbol{\sigma-}\frac{v_{Li}}{E_{Li}}\sigma_{m}\boldsymbol{I}\boldsymbol{,}$ (S2)

where $E_{Li}$ and $v_{Li}$ are Young’s modulus and Poisson’s ratio, respectively, $\boldsymbol{I}$ is the second-order identity tensor. And, the chemical strain $\boldsymbol{\varepsilon}^{c}$ results from the local change in Li concentration

$\boldsymbol{\varepsilon}^{c}=\frac{1}{3}\left( c-c_{0} \right)\Omega_{Li}\boldsymbol{I}.$ (S3)

The viscoplastic behavior of Li is described by the Anand model^[1, 4]^, which captures dislocation-mediated creep. The viscoplastic strain rate ${\dot{\boldsymbol{\varepsilon}}}^{\mathrm{PC}}$ is defined as

${\dot{\boldsymbol{\varepsilon}}}^{\mathrm{PC}}=\frac{3}{2}\varepsilon^{\mathrm{PC}}(\frac{\boldsymbol{\sigma}^{\boldsymbol{'}}}{\bar{\sigma}}),$ (S4)

where $\boldsymbol{\sigma}^{\boldsymbol{'}}\boldsymbol{=\sigma-}\sigma_{m}\boldsymbol{I}$ is the deviatoric stress tensor.

The equivalent plastic shear strain-rate $\varepsilon^{\mathrm{PC}}$ is given by

$\varepsilon^{\mathrm{PC}}=A \exp\left( -\frac{Q}{RT} \right) \left( \frac{\bar{\sigma}}{s} \right)^{m},$ (S5)

where $A$ is a pre-exponential factor, $Q$ is the activation energy, and $s$ is a resistance. With the initial value of $s_{0}$, $s$ evolves as

$s=h_{0}\left| 1-\frac{s}{s^{*}} \right|^{a}\mathrm{sign}\left( 1-\frac{s}{s^{*}} \right)\varepsilon^{\mathrm{PC}},$ (S6)

where positive (negative) $h_{0}$ is the hardening (softening) constant, $a$ is the strain rate sensitivity of hardening (softening), and $s^{*}$ is a saturation stress and can be expressed as

$s^{*}=s\left[ \frac{1}{A}\varepsilon^{\mathrm{PC}}exp\left( \frac{Q}{RT} \right) \right]^{n},$ (S7)

where $s$ is a coefficient, and $n$ is the strain rate sensitivity for the saturation value of $s$.

***Diffusion and Chemo-mechanical Coupling***

Lithium diffusion is governed by the mass balance equation

$\frac{\partial c}{\partial t}=-\nabla\cdot\vec{j}.$ (S8)

The diffusion flux $\vec{j}$ is driven by the gradient of a stress-dependent chemical potential $\mu$

$\vec{j}=-\frac{D_{s}c}{RT}\nabla\mu.$ (S9)

The chemical potential $\mu$ is expressed as

$\mu=\mu_{0}+RT\ln\left( c/c_{0} \right)+\Omega\sigma_{m},$ (S10)

where $\mu_{0}$ is the reference potential.

The stress-dependent chemical potential establishes the coupling from the mechanical field to the diffusion process. Conversely, the concentration variation induced by diffusion produces local volumetric strain as given in Equation S3. This volumetric strain modifies the local stress state through mechanical equilibrium, and thus influences the viscoplastic deformation. Therefore, changes in Li concentration indirectly affect power-law creep behavior by altering the local stress distribution and evolution. This two-way coupling ensures that Li diffusion, stress evolution, and power-law creep are consistently linked through the chemo-mechanical constitutive relations, rather than being treated as independent processes.

***Boundary Conditions and Numerical Implementation***

For the compression problem, no flux of Li atoms is allowed across the boundaries, ensuring that self-diffusion occurs only within the Li layer. The chemical potential gradients responsible for diffusion are generated by the non-uniform hydrostatic stresses induced during constrained compression. The complete set of boundary conditions for the coupled deformation problem is summarized in Table S1.

**Table S1. The complete set of boundary conditions.**

| **Governing Equation** | **Boundary of Lithium** | **Boundary Condition** |
| --- | --- | --- |
| Balance of linear momentum:  $\nabla\cdot\boldsymbol{\sigma}=0$ | Left: $r=0$ | Axisymmetric: $u_{r}=0$ |
|  | Bottom: $z=0$ | Symmetric: $u_{z}=0$ |
|  | Right: $r=R$ | Free surface: $\boldsymbol{\sigma\cdot}\vec{n}=0$ |
|  | Top: z$=H/2$ | $u_{r}=0$ and, $\boldsymbol{\sigma\cdot}\vec{n}=-\sigma_{\mathrm{SP}}\vec{n}$ or $u_{z}=-v_{0}t$ |
| Mass balance equation:  $\frac{\partial c}{\partial t}=-\nabla\cdot\vec{j}$ | All the boundaries: $z=0,H/2$ and $r=0,R$ | No flux:  $\vec{j}\boldsymbol{\cdot}\vec{n}=0$ |

The theoretical framework that couples power-law creep and diffusion is numerically implemented in COMSOL Multiphysics. The *Structural Mechanics* module is used to model the power-law creep behavior of lithium. The *Transport in Solids* interface, in conjunction with the *Shrinkage and Swelling* multiphysics coupling, is utilized to capture stress-driven diffusion and the associated volumetric changes.

The parameters of the model are given in Table S2 and are calibrated against the uniaxial tension experiments on lithium ribbons.^[5]^ The comparison between the experimental data and our numerical results is shown in Figure S1b.

**Table S2. List of parameters used for lithium metal.**

| **Properties** | **Symbol** | **Value** | **Reference** |
| --- | --- | --- | --- |
| Young’s modulus | $E_{Li}$ | 4.9 GPa | ^2, 6^ |
| Poisson’s ratio | $v_{Li}$ | 0.38 | ^2, 6^ |
| Viscoplastic pre-exponential factor | $A$ | 42500 s^-1^ | ^2, 4, 6^ |
| Viscoplastic activation energy | $Q$ | 37 kJ/mol | ^2, 4, 5^ |
| Absolute temperature | $T$ | 298 K | ^2, 4, 5^ |
| Power-low creep exponent | $m$ | 6.6 | ^4, 5^ |
| Deformation resistance saturation coefficient | $s$ | 2 MPa | ^2, 4, 6^ |
| Initial value of the flow resistance | $s_{0}$ | 1.1MPa | ^2, 6^ |
| Hardening constant | $h_{0}$ | 10 MPa | ^2, 4, 6^ |
| Hardening sensitivity | $a$ | 2 | ^2, 4, 6^ |
| Deformation resistance sensitivity | $n$ | 0.05 | ^2, 4, 6^ |
| Diffusion coefficient | $D_{s}$ | $1\times{10}^{-14} m^{2}/s$ | ^7,8^ |
| Gas constant | $R$ | $8.314462 J/(K\cdot mol)$ | --- |
| Molar volume of lithium | $\Omega_{\mathrm{Li}}$ | $1.31\times{10}^{-5} m^{3}/mol$ | --- |
| Equilibrium molar concentration | $c_{0}$ | $7.63\times{10}^{4} mol/m^{3}$ | --- |

**Optimal stack pressure analysis**

Figure S2 illustrates the relationship between optimal stack pressure and lithium layer thickness at different current densities. The stack pressure values are obtained using current density $i$ as an input parameter, and with the applied strain rate calculated via Equation 12. It is note that, as lithium layer thickness $H$ decreases, both the applied equivalent strain rate and aspect ratio $D/H$ increases, leading to a steep rise in the required stack pressure. We also compare our model with experimental data from Haslam et al.^[9]^, which reports that a stack pressure of 3 MPa is required for a lithium anode with diameter $D=6 \mathrm{mm}$ and thickness $H=300 \mu m$ to discharge at a current density of $i=1 mA/cm^{2}$. The experimental results are consistent with our predictions.


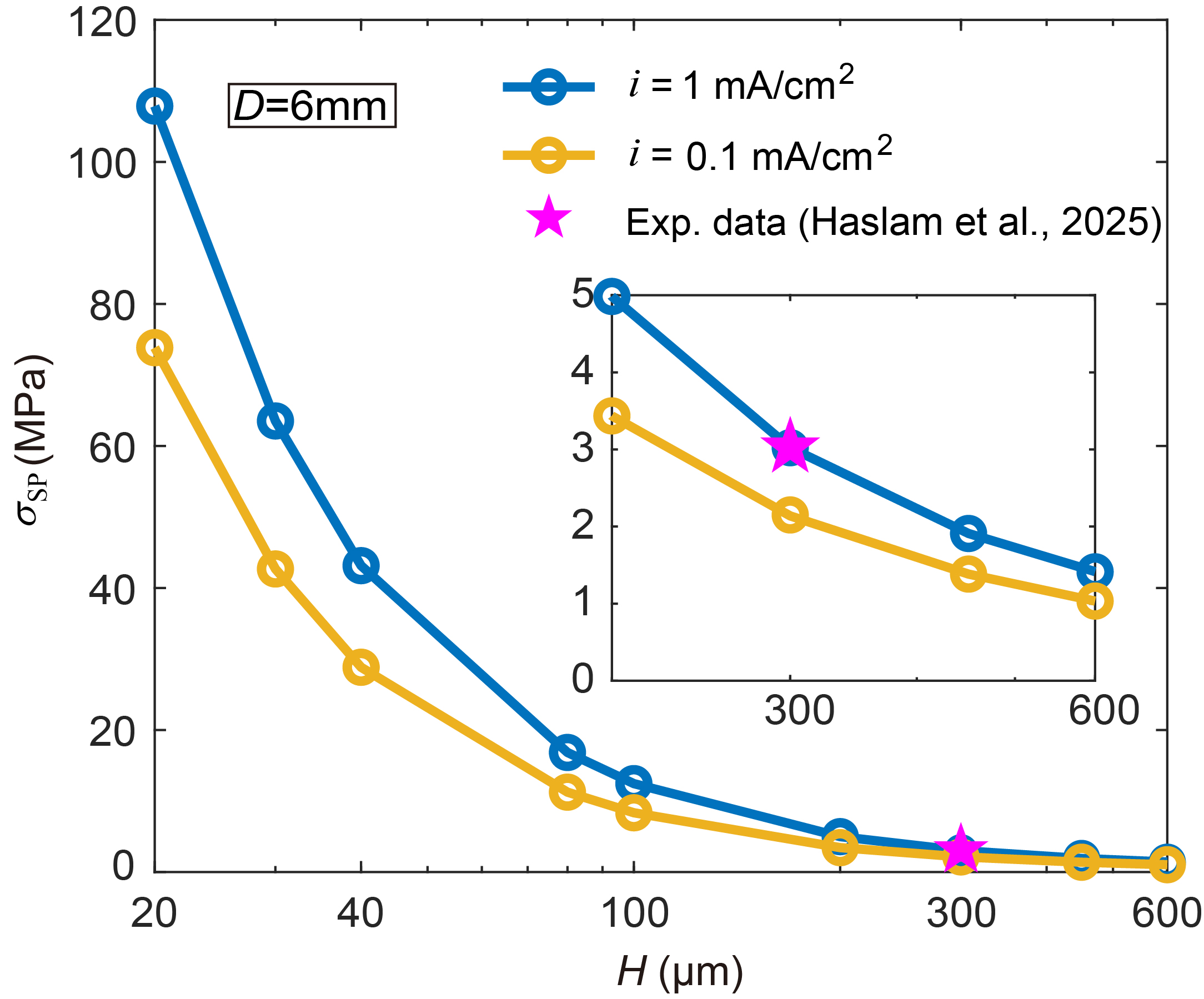


**Figure S2. Stack pressure matching with lithium thickness at different indicated current densities.**

**References**

1. S. Narayan, and L. Anand, “On Modeling the Detrimental Effects of Inhomogeneous Plating-and-Stripping at a Lithium-Metal/Solid-Electrolyte Interface in a Solid-State-Battery,” *Journal of the Electrochemical Society* 167, no. 4 (2020): 040525. https://doi.org/10.1149/1945-7111/ab75c1.

2. Y. Zhao, R. Wang, and E. Martínez-Pañeda, “A Phase Field Electro-Chemo-Mechanical Formulation for Predicting Void Evolution at the Li–Electrolyte Interface in All-Solid-State Batteries,” *Journal of the Mechanics and Physics of Solids* 167 (2022): 104999, https://doi.org/https://doi.org/10.1016/j.jmps.2022.104999.

3. R. Fang, W. Li, J. Jiao, et al., “Modeling the Electro-Chemo-Mechanical Failure at the Lithium-Solid Electrolyte Interface: Void Evolution and Lithium Penetration,” *Journal of the Mechanics and Physics* 192 (2024): 105799. https://doi.org/10.1016/j.jmps.2024.105799.

4. L. Anand, and S. Narayan, “An Elastic-Viscoplastic Model for Lithium,” *Journal of the Electrochemical Society* 166, no. 6 (2019): A1092, https://doi.org/10.1149/2.0861906jes.

5. W. S. LePage, Y. Chen, E. Kazyak, et al., “Lithium Mechanics: Roles of Strain Rate and Temperature and Implications for Lithium Metal Batteries,” *Journal of the Electrochemical Society* 166, no. 2 (2019): A89, https://doi.org/10.1149/2.0221902jes.

6. J. A. Lewis, S. E. Sandoval, Y. Liu, et al., “Accelerated Short Circuiting in Anode-Free Solid-State Batteries Driven by Local Lithium Depletion,” *Advanced Energy Materials* 13, no. 12 (2023): 2204186, https://doi.org/10.1002/aenm.202204186.

7. E. Dologlou, “Self-Diffusion in Solid Lithium,” *Glass Physics and Chemistry* 36, no. 5 (2010): 570. https://doi.org/10.1134/S1087659610050056.

8. H. Yan, K. Tantratian, K. Ellwood, et al., “How Does the Creep Stress Regulate Void Formation at the Lithium-Solid Electrolyte Interface During Stripping?,” *Advanced Energy Materials* 12, no. 2 (2022): 2102283, https://doi.org/https://doi.org/10.1002/aenm.202102283.

9. C. G. Haslam, J. K. Eckhardt, A. Ayyaswamy, et al., “Evaluating Pressure-Dependent Discharge Behavior of Foil Versus in Situ Plated Lithium Metal Anodes in Solid-State Batteries,” *Advanced Energy Materials* 15, no. 12 (2025): 2403614. https://doi.org/https://doi.org/10.1002/aenm.202403614.
